# Effect of Phase-Encoding Direction on Gender Differences: A Resting-State Functional Magnetic Resonance Imaging Study

**DOI:** 10.3389/fnins.2021.748080

**Published:** 2022-01-25

**Authors:** Yun Wang, Xiongying Chen, Rui Liu, Zhifang Zhang, Jingjing Zhou, Yuan Feng, Chao Jiang, Xi-Nian Zuo, Yuan Zhou, Gang Wang

**Affiliations:** ^1^Beijing Key Laboratory of Mental Disorders, National Clinical Research Center for Mental Disorders, Beijing Anding Hospital, Beijing, China; ^2^Advanced Innovation Center for Human Brain Protection, Capital Medical University, Beijing, China; ^3^Beijing Key Laboratory of Learning and Cognition, School of Psychology, Capital Normal University, Beijing, China; ^4^State Key Laboratory of Cognitive Neuroscience and Learning, Beijing Normal University, Beijing, China; ^5^CAS Key Laboratory of Behavioral Science, Institute of Psychology, Beijing, China; ^6^Department of Psychology, University of Chinese Academy of Sciences, Beijing, China

**Keywords:** phase-encoding direction, functional magnetic resonance imaging, spontaneous brain activity, functional connectivity, gender difference

## Abstract

**Aim:**

Neuroimaging studies have highlighted gender differences in brain functions, but conclusions are not well established. Few studies paid attention to the influence of phase-encoding (PE) direction in echo-planar imaging on gender differences, which is a commonly used technique in functional magnetic resonance imaging (fMRI). A disadvantage of echo-planar images is the geometrical distortion and signal loss due to large susceptibility effects along the PE direction. The present research aimed to clarify how PE direction can affect the outcome of a specific research on gender differences.

**Methods:**

We collected resting-state fMRI using anterior to posterior (AP) and posterior to anterior (PA) directions from 113 healthy participants. We calculated several commonly used indices for spontaneous brain activity including amplitude of low frequency fluctuations (ALFF), fractional ALFF (fALFF), regional homogeneity (ReHo), degree centrality (DC), and functional connectivity (FC) of posterior cingulate cortex for each session, and performed three group comparisons: (i) AP versus PA; (ii) male versus female; (iii) interaction between gender and PE direction.

**Results:**

The estimated indices differed substantially between the two PE directions, and the regions that exhibited differences were roughly similar for all the indices. In addition, we found that multiple brain regions showed gender differences in these estimated indices. Further, we observed an interaction effect between gender and PE direction in the bilateral middle frontal gyrus, right precentral gyrus, right postcentral gyrus, right lingual gyrus, and bilateral cerebellum posterior lobe.

**Conclusion:**

These apparent findings revealed that PE direction can partially influence gender differences in spontaneous brain activity of resting-state fMRI. Therefore, future studies should document the adopted PE direction and appropriate selection of PE direction will be important in future resting-state fMRI studies.

## Introduction

Gender differences in behavioral and cognitive domains have been reported by extensive studies (Miller and Halpern, 2014). Males tend to outperform women in mathematics and visual and spatial processing (Rizk-Jackson et al., 2006; Wang et al., 2012), whereas females perform better in verbal skills and memory (Kimura, 1996), facial emotion recognition (Rahman et al., 2004), and emotion processing (Canli et al., 2002). Reports of gender differences have spurred interest in investigating structural and functional brain features, which may underlie previous findings of cognitive and behavioral differences. A meta-analysis of structural brain imaging studies revealed that males exhibit larger brain volumes for the total brain, as well as gray and white matter tissues. Region-specific gender differences have been found in areas such as the frontomedial cortices, amygdala, hippocampus, and insula (Ruigrok et al., 2014). In addition, males tended to exhibit more intrahemispheric connectivity, whereas females appeared to exhibit more interhemispheric connectivity (Ingalhalikar et al., 2014). Apart from these anatomical differences, many studies have reported gender differences in brain functions by utilizing cognitive tasks (Shaywitz et al., 1995; Canli et al., 2002), which showed that male and female brains might have some different neural mechanisms. In addition, the incidence of certain brain disorders, such as autism (Werling and Geschwind, 2013) and major depressive disorder (Albert, 2015), differs between genders. Thus, researches into the neurobiology of gender differences may provide insights into the risk and protective factors associated with psychopathology (Cahill, 2006).

In recent decades, resting-state functional magnetic resonance imaging (rsfMRI) has become a favorable and valuable research modality for neuroimaging studies, because it is more acceptable and prevents possible task bias compared with task-based fMRI (Biswal, 2012; Yang et al., 2020). rsfMRI involves analysis of spontaneous brain function using blood oxygen level dependent (BOLD) contrasts, such as amplitude of low frequency fluctuations (ALFF), fractional ALFF (fALFF), regional homogeneity (ReHo), degree centrality (DC), and resting-state functional connectivity (rsFC), which has provided a new venue to understand the brain’s intrinsic functional organization (Fox and Raichle, 2007; Deco et al., 2011; Rosazza and Minati, 2011). Neuroimaging studies utilizing different types of analytical methods have reported differences in brain activity between males and females, but conclusions regarding gender effects are not well established. Xu et al. (2015) reported that males showed higher ReHo in the left precuneus, while females showed higher ReHo in the right middle cingulate gyrus, fusiform gyrus, left inferior parietal lobule, precentral gyrus, supramarginal gyrus, and postcentral gyrus. In comparison, Dai et al. (2012) found that males showed significantly higher ReHo in the left occipital lobe, and left temporal lobe, left frontal lobe, and lower ReHo in the right insula and in the left parietal lobe compared with females. Bluhm et al. (2008) detected stronger functional connectivity (FC) for females within the posterior cingulate cortex/precuneus and the bilateral medial prefrontal cortex. In addition, Biswal et al. (2010) found consistent gender variations of FC using three distinct methods (seed-based, fALFF, and independent component analyses), whereas Weissman-Fogel et al. (2010) found no significant gender differences in FC. Besides, Filippi et al. (2013) reported stronger female FC for frontal and temporal lobes, but Zhang et al. (2016) showed the opposite trend (Zhang et al., 2016). We can see that the findings of previous studies remain inconclusive and in some cases contradictory. Many confounding factors may cause the inconsistency of findings in these researches, such as sample size, sample heterogeneity, parameters of image data, calculation process, statistical power, and so on. Among them, an important and easily overlooked factor is the phase-encoding (PE) direction of echo-planar imaging (EPI) when collecting fMRI data.

**FIGURE 1:**
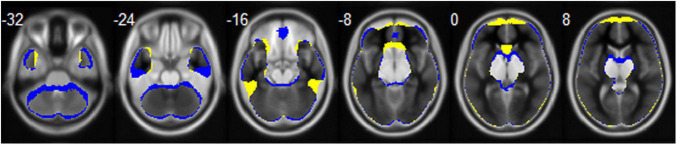
The mean whole brain mask calculated based on the functional images of all the participants for the AP (colored in blue) and PA (colored in yellow) PE direction, respectively. Brain regions that the two directions overlapped are displayed in gray.

Echo-planar imaging is a commonly used technique and plays a crucial role in fMRI (Stehling et al., 1991). However, a major disadvantage of acquiring data with EPI is the presence of susceptibility to magnetic field inhomogeneity, resulting in geometrical distortion and signal loss (Jezzard and Balaban, 1995). Previous studies have shown that EPI can cause signal loss in brain areas close to the air/tissue boundary, such as the orbitofrontal cortex (OFC) and the temporal gyrus (TG; Ojemann et al., 1997; Lipschutz et al., 2001). EPI images with anterior-to-posterior (AP) or posterior-to-anterior (PA) PE directions are most frequently applied in fMRI of the brain. Meanwhile, it is known that geometrical distortion and signal loss of EPI images is related to PE direction and this effect is prominent in the OFC and TG (De Panfilis and Schwarzbauer, 2005; Weiskopf et al., 2006). The mean whole brain mask calculated based on the functional images of all the participants with opposite PE directions in our study is shown in Figure 1. We can see that the AP image and PA image are quite different. The AP image exhibits greater signal loss in the OFC, middle TG, and anterior cingulate gyrus, and the PA image exhibits greater signal loss in the gyrus rectus, inferior TG, caudate, and cerebellum of the brain. To correct for EPI distortions, it is recommended the use of correction methods, such as Field-Map, TOPUP, TISAC, and S-Net, in fMRI data analysis (Togo et al., 2017; Duong et al., 2020a,b). More importantly, the brain areas showing gender differences in previous studies involved the regions that can be affected by PE direction, such as temporal and frontal lobe. Despite this, the majority of fMRI studies have not reported the PE direction. Therefore, investigating this potential effect of PE direction on spontaneous brain activity is particularly important because such kind of image artifact could affect the conclusions on gender differences in brain functions.

Based on the above reasoning, we hypothesized that gender differences in spontaneous brain activity may differ substantially depending on PE direction. We collected two sets of rsfMRI imaging data with opposite polarities of the PE direction (i.e., AP and PA) from healthy participants and investigated whether the outcome of gender differences in spontaneous brain activity was influenced by PE direction. We utilized spontaneous brain activity indices (i.e., ALFF, fALFF, ReHo, DC, and seed-based rsFC), which are the methods performed most frequently by the studies when analyzing rsfMRI data (Biswal et al., 2010; Seidel et al., 2020).

## Materials and Methods

### Participants

One hundred thirteen healthy participants were recruited from Beijing Anding Hospital of Capital Medical University. Demographic details are shown in Table 1. Participants were recruited by advertisements from local communities. They had no history of psychiatric illness or substance abuse and no history of psychotic disorders among their first-degree relatives. Exclusion criteria for participants included a history of head trauma, neurological illness, and serious medical or surgical illness. After receiving a complete description of the study, all participants gave their written informed consent. This study was approved by the Ethics Committee of Beijing Anding Hospital of Capital Medical University.

**TABLE 1:** Demographic data.

Demographic category	Male *N* = 43	Female *N* = 70	*t*	*p*
Age (years)	27.37 ± 7.04	26.33 ± 4.40	0.971	0.334
Education			5.439	0.066
High school	6	2		
Undergraduate	22	45		
Graduate	15	23		
Mean FD				
AP	0.145 ± 0.076	0.132 ± 0.061	1.012	0.314
PA	0.137 ± 0.066	0.126 ± 0.054	0.958	0.340

*AP, anterior to posterior; PA, posterior to anterior; FD, frame-wise displacement.*

### Image Acquisition

Each participant completed two rsfMRI scans and a high-resolution structural scan. MRI data were collected using a Siemens 3.0 Tesla whole-body scanner in Beijing Anding Hospital. Subjects were instructed to lie still inside the scanner, close their eyes, stay awake, and try not to think about anything special. Two transverse EPI images were acquired with opposite polarities of the PE direction (i.e., AP and PA) for each participant, parallel to the AC–PC line. The two sets of fMRI scans were acquired in the same order: PA PE direction before AP PE direction. For each direction, 200 volumes of fMRI images were acquired axially using the following parameters: repetition time (TR) = 2000 ms, echo time (TE) = 30 ms, field of view (FOV) = 200 mm, voxel size = 3.13 mm × 3.13 mm × 4.2 mm, matrix = 64 × 64, flip angle (FA) = 90°, number of slices = 33, slice thickness = 3.5 mm, slice spacing = 0.7 mm. A sagittal T1-weighed structural scan (TR = 2.53 s, TE = 1.85 ms, matrix = 256 × 256, FOV = 256 mm, slice thickness = 1 mm, FA = 9°) was acquired in order to co-register it with the fMRI data. Simultaneously, we collected spin-echo EPI images for diffusion MRI in opposite PE directions (i.e., AP and PA) for each participant (TR = 9.2 s, TE = 78 ms, matrix = 128 × 128, FOV = 256 mm, voxel size = 2 mm × 2 mm × 2 mm, echo train length = 48, slice thickness = 2 mm, slice spacing = 2 mm, number of slices = 75, FA = 90°).

### Data Preprocessing

Resting-state fMRI data was first corrected for susceptibility distortion *via* FSL’s ‘‘TOPUP’’ tool. We used reversed PE distortion field maps collecting from diffusion MRI (*b* = 0 s/mm^2^) to correct fMRI images. The scan parameters of the diffusion MRI image were not consistent with the fMRI image; thus, the diffusion MRI images were first registered to fMRI images. Then, the fMRI images were corrected by running topup and then applytopup. Next, the corrected fMRI images were preprocessed by using the Data Processing Assistant for Resting-State fMRI (DPARSFA 4.4)^1^ (Yan and Zang, 2010), which is based on the Statistical Parametric Mapping (SPM) program^2^ and the Resting-State fMRI Data Analysis Toolkit (see text footnote 1) (Song et al., 2011). Prior to preprocessing, the first 5 volumes were discarded to allow for signal stabilization. The remaining volumes acquired from each subject were corrected for the differences in slice acquisition times. The resultant images were then realigned to correct for small movements that occurred between scans. Following motion correction, each participant’s BOLD fMRI data were co-registered to the participant’s anatomical images. Then, each participant’s anatomical images were segmented into gray matter, white matter, and cerebral spinal fluid (CSF) images, and the deformation fields were derived to transform each participant’s BOLD fMRI data into the Montreal Neurological Institute (MNI) standard space. Lastly, 24 head motion parameters (Friston et al., 1996), the first five principle components of signals from white matter and CSF, polynomial trend, and head motion scrubbing regressors were regressed out for every voxel using linear regression. The resulting maps were then registered into MNI space with 2 mm × 2 mm × 2 mm cubic voxels using the transformation information acquired from T1 image unified segmentation. A smoothing kernel of 4 mm was applied after registration (except for ReHo and DC). Finally, temporal filtering (0.01–0.1 Hz) of the time series was performed (except for ALFF and fALFF). Participants with acceptable head motion were included for further analysis. First, participants whose head motion was larger than 3 mm in the x, y, or z direction or had more than 3° of angular rotation will be excluded. Second, mean frame-wise displacement (FD), which considers the measures of voxel-wise differences in motion in its derivation (Jenkinson et al., 2002), was used as a measure of the micro-head motion of each subject (Yan et al., 2013a). Participants whose mean FD was larger than 3 interquartile ranges from the sample median or had less than 100 “good” volumes of data (FD threshold ≤0.5 mm) will be excluded from further analysis. No participant was excluded according to the motion threshold set for the study.

### Spontaneous Activity Indices

We calculated ALFF, fALFF, ReHo, and DC values and seed-based FC for each participant by using DPARSFA 4.4.

Amplitude of low frequency fluctuations reflects the strength or intensity of low frequency oscillations (Zang et al., 2007; Zuo et al., 2010). fALFF, as an improved measure, is defined as the ratio of total amplitude within the low-frequency range to the total amplitude of the entire detectable frequency range (Zou et al., 2008). After preprocessing, the ALFF/fALFF values of each subject were calculated in a voxel-wise way. First, the power spectrum was acquired by using fast Fourier transformation to convert each voxel’s time series into frequency domain. Then, each frequency of the power spectrum was squared root transformed at each voxel. The averaged squared root of the frequency range of 0.01–0.1 Hz was defined as the ALFF value. fALFF is defined as the division of ALFF within the specified frequency band (0.01–0.1 Hz) by the entire frequency range observed in the signal. Finally, subject-level voxel-wise ALFF/fALFF maps were standardized into subject-level z-score maps.

Regional homogeneity estimation was done on a voxel-by-voxel basis by calculating Kendall’s coefficient of concordance, which estimates similarity in the time series of a given voxel to its nearest 26 voxels (Zang et al., 2004; Jiang and Zuo, 2016). Finally, the subject-level voxel-wise ReHo maps were standardized into subject-level z-score maps and a smoothing kernel of 4 mm was applied.

Degree centrality maps were generated in a voxel-wise fashion. First, the preprocessed functional images were subjected to voxel-based whole-brain correlation analysis. The time course of each voxel from each participant was correlated with the time course of every other voxel, which resulted in a correlation matrix. An undirected adjacency matrix was then obtained by thresholding each correlation at *r* < 0.25 (Buckner et al., 2009; Zuo et al., 2012; Yan et al., 2013b,c). Then, the DC was computed as the number of significant correlations (binarized) or as the sum of the weights of the significant connections (weighted) for each voxel (Zuo et al., 2012). Finally, the subject-level voxel-wise DC was converted into a z-score map (Zuo et al., 2012; Yan et al., 2013c) and a smoothing kernel of 4 mm was applied.

Seed-based rsFC analysis was conducted by extracting the time series from the posterior cingulate cortex (PCC) region. The seed was constructed using a radius 10-mm sphere located at (0, −53, 26) in MNI space, in accordance with previous studies (Andrews-Hanna et al., 2007; Ward et al., 2014; Lin et al., 2017). First, the time series of each voxel within the seed were extracted. Second, the time series of each voxel in the seed region were averaged to acquire the mean time series of the seed. Third, Pearson’s correlation coefficients between the mean time series of the seed and the time series of each voxel within the whole brain were calculated (Uddin et al., 2009). The Pearson’s correlation coefficients were used to construct each subject’s rsFC map. Finally, the rsFC maps were converted into z-score maps, which were included in the second-level analysis.

### Statistical Analysis

Second-level analyses for the above-mentioned indices were performed by using SPM12 (Wellcome Department of Cognitive Neurology, London, United Kingdom). We performed 2 (gender) × 2 (PE direction) flexible design to test the following three group comparisons: (i) AP versus PA; (ii) male versus female; and (iii) the interaction between PE direction and gender. In these analyses, age, education level, and mean FD were imported as covariates. Statistical significance was set at voxelwise *p* < 0.001 in conjunction with clusterwise family-wise error (FWE) *p* < 0.05 to correct for multiple comparisons. These second-level analyses of group comparisons were limited within a premade gray mask, which was the intersection of all the subjects’ mean gray masks calculated based on their T1 images and the mean whole brain mask calculated based on the functional images.

### Validation Analysis

We performed the same statistical analysis to the Human Connectome Project (HCP) data to validate our hypothesis. Specifically, we obtained minimally preprocessed resting-state fMRI data from 99 unrelated participants and these data included opposite polarities of the PE direction for each participant. There were 46 males and 53 females, and the age range of the participants was from 22 to 35 years old. The PE directions of the HCP data were left-to-right (LR) or right-to-left (RL), which are different from the data we collected. The preprocessing pipeline of volume-based analyses of the HCP fMRI data includes removing spatial distortions, realigning volumes to compensate for subject motion, registering the fMRI data to the structural images, reducing the bias field, normalizing the 4D image to a global mean, and masking the data with the final brain mask (Glasser et al., 2013). Based on the minimally preprocessed data, we calculated ALFF, fALFF, ReHo, DC, and rsFC of the PCC for each participant and for each PE direction. Then, we performed 2 (gender) × 2 (PE direction) flexible design in SPM software to test the interaction between PE direction and gender. The statistical significance level was set at voxelwise *p* < 0.001 in conjunction with clusterwise FWE correction *p* < 0.05.

## Results

### Anterior to Posterior Versus Posterior to Anterior

**FIGURE 2:**
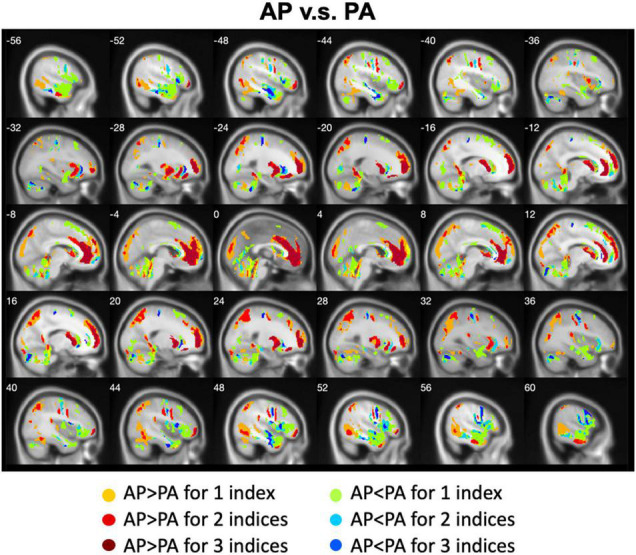
Overlapped regions that showed different local spontaneous brain activity (i.e., ALFF, fALFF, and ReHo) depending on PE direction (AP vs. PA). Dark red indicates common regions for all the three indices where the AP direction exhibited significantly higher brain activity than the PA direction. Light red indicates common regions for any two indices where the AP direction exhibited significantly higher brain activity than the PA direction. Dark blue and light blue indicate the reverse.

**FIGURE 3:**
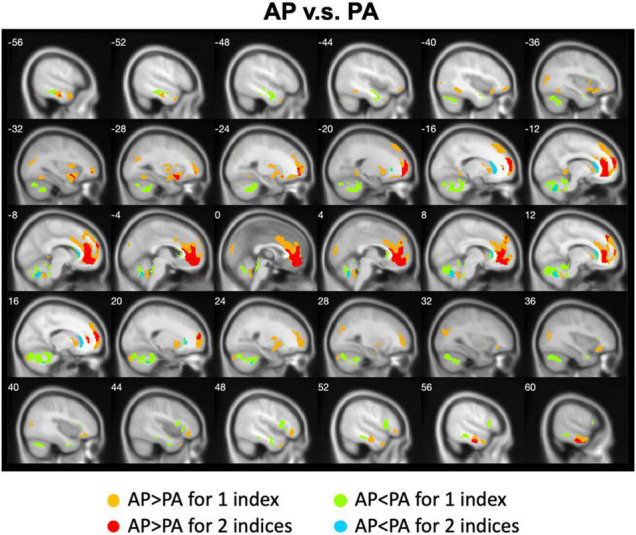
Overlapped regions that showed different global brain connectivity (i.e., DC and rsFC of PCC) depending on PE direction (AP vs. PA). Light red indicates common regions for the two indices where the AP direction exhibited significantly higher FC than the PA direction. Light blue indicates the reverse.

We calculated the differences between AP imaging and PA imaging for each index across all participants. Please see the Supplementary Figures 1–5. We further overlapped these images in order to show the common regions. Generally, ALFF, fALFF, and ReHo reflect local spontaneous brain activity, while DC and rsFCs reflect global brain connectivity. Therefore, we distinguished these two types of indices to show the overlapped brain areas. Compared with PA imaging, we found that AP imaging exhibited greater local spontaneous brain activity (i.e., ALFF, fALFF, and ReHo) in the anterior cingulate cortex, medial frontal cortex, superior frontal gyrus, middle frontal gyrus (MFG), inferior orbitofrontal gyrus, putamen, caudate, insula, inferior parietal lobule, superior parietal lobule, and parahippocampal gyrus, but showed weaker local spontaneous brain activity in the medial orbitofrontal cortex, inferior frontal gyrus, parts of caudate and putamen, precentral gyrus, middle TG, and cerebellum (Figure 2). As to the rsFC of PCC, AP imaging exhibited greater rsFCs in the anterior cingulate cortex, medial frontal cortex, superior frontal gyrus, and inferior TG, but weaker rsFCs in the caudate and cerebellum when compared with PA imaging. Similar patterns were also found for the DC values (Figure 3).

### Male Versus Female

**FIGURE 4:**
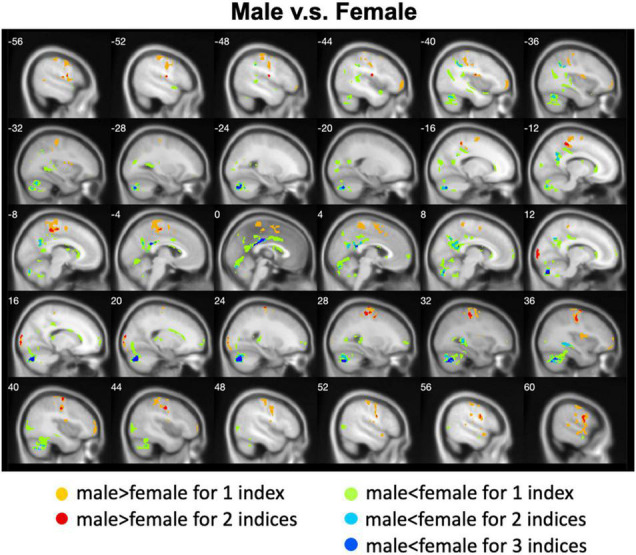
Overlapped regions that showed different local spontaneous brain activity (i.e., ALFF, fALFF, and ReHo) between males and females. Light red indicates common regions for any two indices where the males exhibited significantly higher brain activity than the females. Light blue indicates the reverse. Dark blue indicates common regions for all the three indices where the females exhibited significantly higher brain activity than the males.

**FIGURE 5:**
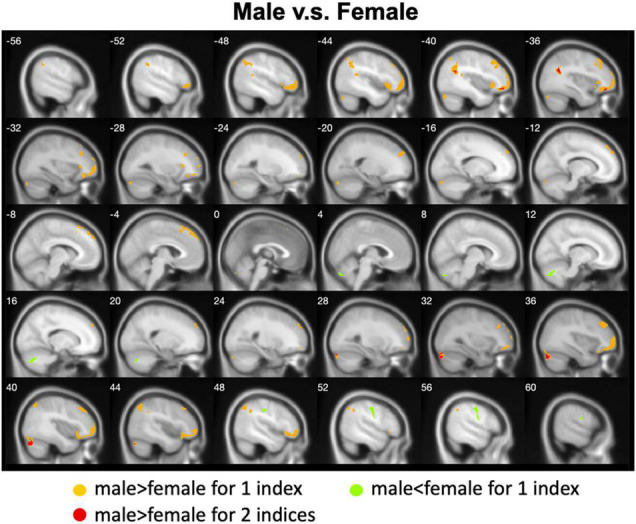
Overlapped regions that showed different global brain connectivity (i.e., DC and rsFC of PCC) between males and females. Light red indicates common regions for the two indices where the males exhibited significantly higher rsFC than the females.

We calculated gender differences for each index (Tables 2, 3 and Supplementary Figures 6–10). We also overlapped these images in order to show the common regions. The results of the male-versus-female comparisons can be seen in Figures 4, 5. Compared with females, we found males exhibited greater local spontaneous brain activity (i.e., ALFF, fALFF, and ReHo) in bilateral precentral gyrus, postcentral gyrus, left paracentral lobule, right cuneus, and medial frontal gyrus, but showed weaker local spontaneous brain activity in bilateral precuneus, posterior and middle cingulate gyrus, left inferior parietal lobule, right hippocampus, and bilateral cerebellum posterior lobe (Figure 4). As to the rsFC of PCC, males exhibited greater rsFCs with the left MFG, left angular gyrus, left superior TG, and right cerebellum posterior lobe when compared with females. Similar patterns were also found for the DC values (Figure 5).

**TABLE 2:** Regions showing significant gender differences (male > female) for each index.

Brain region	Hemisphere	BA	MNI coordinates	Peak *T* value	Cluster size
**ALFF**					
Middle temporal gyrus/superior temporal gyrus	Left	22	−64, −50, 8	5.09	86
Precentral gyrus/postcentral gyrus	Left	6/4	−60, −4, 40	5.23	424
Superior temporal gyrus	Right	22/42	66, −34, 10	7.66	134
Precentral gyrus/postcentral gyrus	Right	6/3	50, −20, 60	6.95	1066
Postcentral gyrus/insula	Left	43/13	−44, −12, 18	5.15	149
Postcentral gyrus	Left	40/2	−38, −32, 50	5.23	109
Paracentral lobule/medial frontal gyrus	Bilateral	6	−10, −34, 54	6.33	791
Medial frontal gyrus	Bilateral	6	−8, −4, 58	6.85	331
Postcentral gyrus	Left	2/3	−56, −28, 52	5.21	173
Precentral gyrus	Left	4	−34, −22, 62	4.87	73
**fALFF**					
Precentral gyrus/superior temporal gyrus	Right	6	54, 10, −12	5.10	333
Cuneus/middle occipital gyrus	Right	18	24, −100, 2	5.13	124
Precentral gyrus/insula	Left	6/13	−44, −10, 18	5.58	235
Insula	Right	13	34, 6, 14	4.75	130
Precentral gyrus	Right	6	30, −14, 70	5.00	390
**ReHo**					
Cuneus/middle occipital gyrus	Right	18/17	16, −100, 0	5.49	213
Middle frontal gyrus	Left	10/11	−42, 56, −4	5.22	166
Middle frontal gyrus	Right	10/11	42, 58, −2	4.62	101
Paracentral lobule/medial frontal gyrus/precuneus	Left	6/5/4	−10, −36, 54	5.14	97
**DC**					
Cerebellum posterior lobe	Right		36, −82, −28	5.25	163
Cerebellum posterior lobe	Left		−42, −78, −28	4.87	132
Middle frontal gyrus/inferior frontal gyrus	Right	10/11/47	40, 58, −6	6.49	608
Middle frontal gyrus/inferior frontal gyrus/superior frontal gyrus	Left	10/8/47/9	−38, 60, 2	6.64	1267
Angular gyrus/inferior parietal lobule	Left	40/39	−40, −54, 34	5.62	339
Middle frontal gyrus/superior frontal gyrus	Right	9	40, 36, 44	4.93	247
Middle frontal gyrus	Left	9/8	−30, 32, 34	4.65	224
Angular gyrus/inferior parietal lobule	Right	40/7	44, −64, 50	5.33	219
**PCC_FC**					
Cerebellum posterior lobe	Right		40, −80, −30	4.71	170
Middle frontal gyrus/inferior frontal gyrus	Left	11/47	−36, 34, −12	5.47	140
Superior temporal gyrus/angular gyrus	Left	13	−36, −56, 24	4.68	138

**TABLE 3:** Regions showing significant gender differences (female > male) for each index.

Brain region	Hemisphere	BA	MNI coordinates	Peak *T* value	Cluster size
**ALFF**					
Cerebellum posterior lobe	Left		−18, −80, −40	5.94	905
Cerebellum posterior lobe	Right		8, −50, −44	5.79	229
Cerebellum posterior lobe	Right		22, −76, −38	6.27	2038
Caudate	Bilateral		−8, −4, 16	4.73	150
Posterior cingulate/precuneus	Bilateral	31/23	0, −56, 16	5.13	452
Precuneus	Right	7	10, −62, 36	4.79	86
**fALFF**					
Cerebellum posterior lobe	Left		−36, −64, −26	5.36	492
Cerebellum posterior lobe	Right		18, −72, −36	5.61	796
Hippocampus	Right		34, −38, 2	5.73	102
Superior frontal gyrus	Right	10	4, 68, 0	5.41	190
Cingulate gyrus	Bilateral	23	2, −26, 34	5.43	173
Inferior parietal lobule	Left	40	−36, −50, 42	6.09	134
**ReHo**					
Cerebellum posterior lobe	Right		22, −74, −36	5.96	357
Cerebellum posterior lobe	Left		−24, −76, −36	4.37	109
Middle temporal gyrus	Right	21	70, −22, −8	5.62	124
Superior temporal gyrus	Left	38	−52, 6, −8	4.89	103
Hippocampus/caudate	Left		−34, −44, 0	6.28	276
Hippocampus/caudate	Right		34, −36, 0	6.30	427
Middle occipital gyrus	Right	19	54, −72, 4	4.37	207
Caudate	Left		−16, 32, 4	4.69	131
Cuneus/middle occipital gyrus	Left	18/17	−18, −82, 14	5.69	219
Caudate	Right	18/17	20, 26, 14	5.43	181
Middle temporal gyrus	Left	39	−36, −60, 16	5.72	173
Superior temporal gyrus	Left	13	−46, −38, 6	4.62	152
Cingulate gyrus/precuneus	Bilateral	31/23	−16, −50, 30	6.12	1406
Inferior parietal lobule	Left	40	−38, −40, 36	4.33	108
Cingulate gyrus	Right	31	14, −30, 40	5.64	203
**DC**					
Cerebellum posterior lobe	Right		16, −72, −36	4.56	167
Precentral gyrus/postcentral gyrus	Right	4/3	56, −8, 24	4.18	161

### Interaction Between Gender and Phase-Encoding Direction

**FIGURE 6:**
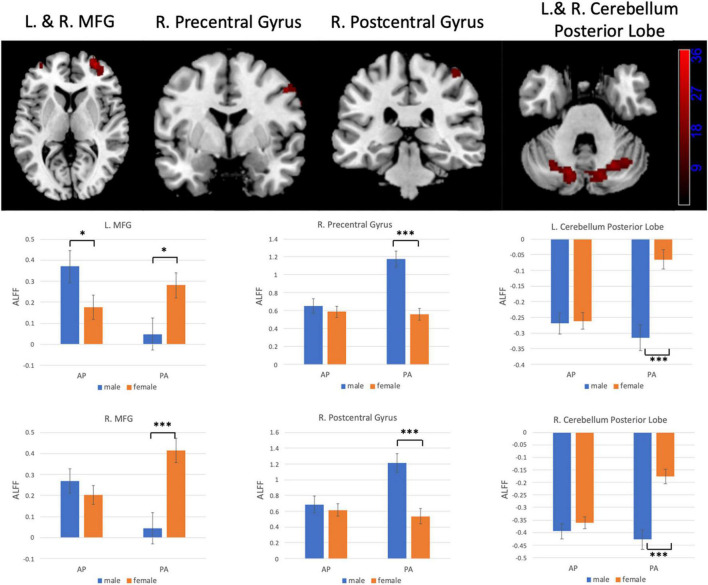
Regions influenced by the interaction effect between gender and PE direction (AP vs. PA) for ALFF and the results of *post hoc* analysis. MFG, middle frontal gyrus. *p < 0.05, ^***^p < 0.001.

**FIGURE 7:**
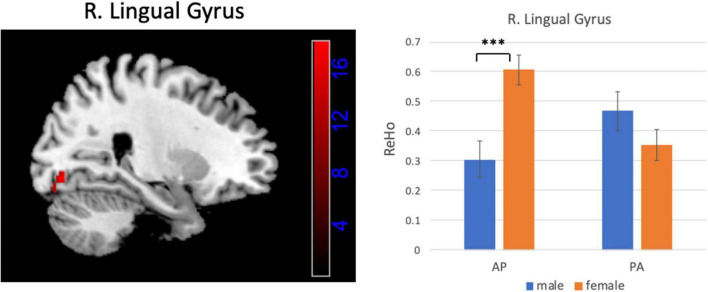
Brain region influenced by the interaction effect between gender and PE direction (AP vs. PA) for ReHo and the result of *post hoc* analysis. ^***^p < 0.001.

Figures 6, 7 show the interaction effect between gender and PE direction for ALFF and ReHo, respectively. Specifically, we found that gender differences in ALFF differed substantially in the bilateral MFG, right precentral gyrus, right postcentral gyrus, and bilateral cerebellum (posterior lobe) depending on PE direction (Table 4). In the left MFG, males showed significantly higher ALFF values for AP imaging (*p* = 0.045) but significantly lower ALFF values for PA imaging (*p* = 0.019) than females. In the right MFG, males showed significantly lower ALFF value than females for PA imaging (*p* < 0.001), but no gender difference was found for AP imaging (*p* = 0.371). In the right precentral and postcentral gyri, males showed significantly higher ALFF values than females for PA imaging (both *p* < 0.001), whereas no gender difference was found for AP imaging (both *p* > 0.05). In the bilateral cerebellum posterior lobe, males showed significantly lower ALFF values than females for PA imaging (both *p* < 0.001), whereas no gender difference was found for AP imaging (both *p* > 0.05, Figure 6). In addition, gender differences in ReHo differed considerably in the right lingual gyrus depending on PE direction (Table 4); females showed significantly higher ReHo than males for AP imaging (*p* < 0.001), but no gender difference was found for PA imaging (*p* = 0.17, Figure 7). No substantial interaction effect was found in the other indices.

**TABLE 4:** Regions influenced by the interaction effect between gender and PE direction (AP vs. PA) for ALFF and ReHo.

Brain region	Hemisphere	BA	MNI coordinates	Peak *T* value	Cluster size
**ALFF**					
Middle frontal gyrus	Left	10	−30, 54, 12	20.51	56
Middle frontal gyrus	Right	10	40, 46, 0	36.48	460
Precentral gyrus	Right	6	64, −4, 36	29.87	65
Postcentral gyrus	Right	3	42, −30, 62	17.91	64
Cerebellum posterior lobe	Left		−16, −72, −30	27.86	333
Cerebellum posterior lobe	Right		26, −66, −32	23.04	405
**ReHo**					
Lingual gyrus	Right	18	30, −84, −16	17.78	68

### Validation Analysis

In the HCP data, we also found remarkable interaction effect between gender and PE direction for all the indices except the rsFC of PCC. Specifically, we observed an interaction effect between gender and PE direction in the bilateral frontal gyrus, bilateral temporal gyrus, left putamen, left parahippocampal gyrus, and bilateral cerebellum (posterior lobe) depending on specific spontaneous brain functional indices (Table 5). Detailed results of *post hoc* analysis can be seen in the Supplementary Figures 11–14.

**TABLE 5:** Regions influenced by the interaction effect between gender and PE direction (LR vs. RL) for each index in the HCP data.

Brain region	Hemisphere	BA	MNI coordinates	Peak *T* value	Cluster size
**ALFF**					
Putamen/insula/inferior frontal gyrus	Left	13	−32, 24, 4	40.69	677
Putamen	Left		−38, −24, −4	25.50	80
Superior frontal gyrus/middle frontal gyrus	Left	8	−20, 36, 44	28.10	124
Superior frontal gyrus	Right	8	14, 44, 46	29.53	120
Inferior temporal gyrus	Left	20	−54, −30, −30	21.41	120
Inferior temporal gyrus	Right	20	60, −36, −28	23.62	98
Cerebellum posterior lobe	Left		−52, −70, −42	23.88	90
**fALFF**					
Superior frontal gyrus/middle frontal gyrus	Left	9	−30, 38, 32	22.68	51
Superior frontal gyrus/middle frontal gyrus	Left	8/9	−20, 38, 44	21.99	51
Inferior parietal lobule	Right	40	56, −42, 32	22.26	90
Parahippocampal gyrus	Left	34	−14, −8, −20	19.66	73
**ReHo**					
Inferior frontal gyrus/superior temporal gyrus/middle frontal gyrus/insula	Left	47	−36, 12, −26	29.98	650
Superior temporal gyrus	Right	38	38, 20, −26	22.48	121
Orbital part of superior frontal gyrus	Left	11	−10, 58, −22	29.23	127
Orbital part of superior frontal gyrus	Right	11	10, 60, −26	21.08	85
Parahippocampal gyrus	Left	28	−14, −12, −20	28.97	185
Superior frontal gyrus/middle frontal gyrus	Left	9	−22, 40, 30	30.35	396
Cerebellum posterior lobe	Right		22, −62, −58	26.36	199
Cerebellum posterior lobe	Right		38, −68, −46	19.27	80
Cerebellum posterior lobe	Right		12, −84, −38	27.99	156
**DC**					
Superior frontal gyrus/middle frontal gyrus	Left	9/10	−30, 42, 30	22.36	113
Superior frontal gyrus/medial frontal gyrus	Left	10	−20, 58, 0	22.56	50
Superior frontal gyrus/medial frontal gyrus	Left	10/9	−4, 60, 28	25.31	58
Superior temporal gyrus/postcentral gyrus/precentral gyrus	Left	42/41	−56, −28, 6	23.22	55

## Discussion

It is known that susceptibility-induced imaging artifacts can affect functional image quality, but it was unclear to what extent the PE direction matters for gender differences in brain functional estimates. Here, we used a large set of functional images with opposite PE directions (AP and PA) to investigate the effects of PE directions on commonly used spontaneous brain functional indices and on gender differences in these indices. We also validated our hypothesis by applying the HCP data.

We found that spontaneous brain functional indices showed remarkable differences between the AP and PA PE directions. Previous studies indicated that signal loss differs in the OFC and TG when comparing functional images of AP and PA PE directions. Thus, we can speculate that this difference in signal loss between the two PE directions could also have impact on the current calculated indices. However, in our study, we found a more extensive brain area than that for signal loss when comparing the differences in spontaneous brain functional indices between AP and PA PE directions. This indicates that the influence of PE direction on spontaneous brain functional indices is more widespread than expectation. One previous study also found the similar pattern by investigating large-scale FC based on independent component analysis (ICA) in a small sample clinical population (Mori et al., 2018). They found that AP imaging exhibited greater FC in the anterior cingulate cortex, medial prefrontal cortex, medial orbitofrontal cortex, subcallosal cortex, thalamus, posterior middle TG, and temporoparietal junction; PA imaging exhibited greater FC in the temporal pole, anterior middle TG, lateral orbital-frontal cortex, fusiform gyrus, cuneal cortex, and lingual cortex. Overall, the differences between the two directions in that study are also mainly concentrated in the brain areas such as the anterior cingulate cortex, medial prefrontal cortex, OFC, and TG, similar to our findings. Therefore, when reporting the results of a rsfMRI experiment, researchers should specify the polarity of the PE direction that they use in order to provide enough reference information for future studies.

We investigated gender differences in these spontaneous brain functional indices and found that multiple brain regions showed gender differences in these estimated indices. Most studies that note gender differences tend to report a mix of findings with greater spontaneous brain activity for either males or females (Biswal et al., 2010; Tomasi and Volkow, 2012; Xu et al., 2015), just like the findings in our study. As specific results varied across previous studies, not all of our results are consistent with those reported in the published work. For example, in our study, we found that females exhibited significantly higher ReHo in the bilateral TG, occipital gyrus, caudate, cingulate gyrus, cerebellum posterior lobe, and left inferior parietal lobule and lower ReHo in the bilateral MFG, right cuneus, and left paracentral lobule when compared with males. Xu et al. (2015) reported that females showed higher ReHo in the right middle cingulate gyrus, fusiform gyrus, left inferior parietal lobule, precentral gyrus, supramarginal gyrus, and postcentral gyrus in a large healthy population, while Dai et al. (2012) only showed difference in the right insula and left parietal lobe in a relatively small sample. We can see that the regions showing gender differences in ReHo in our study and previous studies have subtle differences. As to the DC, one previous study found gender effects for long- and short-range functional connectivity density (FCD), an index similar to DC. Specifically, females had higher FCD in the default mode network (posterior cingulate, ventral precuneus, angular gyrus, and ventral prefrontal cortex) and lower FCD in the somatosensory and motor cortex than males (Tomasi and Volkow, 2012). In our study, we also found that males showed higher DC in the prefrontal cortex and angular gyrus and lower DC in the precentral and postcentral gyri, which is consistent with previous findings (Tomasi and Volkow, 2012). For the rsFC of PCC, we found that males exhibited significantly higher connections with the left MFG, left superior TG, and right cerebellum. These findings are consistent with a previous study, in which males exhibited greater FC than females and the differences were mainly present in frontal, parietal, and temporal lobes in a large sample (Biswal et al., 2010). However, we did not find that females have any significantly higher connections than males, which is different from this study (Biswal et al., 2010). Previous studies have rarely studied gender differences for the other indices (ALFF and fALFF), yet gender differences in these indices in the current study were also found to be mainly present in the frontal and temporal lobe, precentral and postcentral gyri, and cerebellum. It is worth noting that the brain regions showing gender differences in our research were found across the two PE directions. This suggests that these findings in our study are independent of PE direction.

Finally, we investigated the interaction between gender and PE direction to exemplify how PE directions (AP and PA) influence the results of gender differences. We found that the results of male-versus-female comparisons were much different depending on the PE directions in several brain regions. Specifically, we found that the patterns of gender differences in ALFF differed considerably in the bilateral MFG, right precentral gyrus, right postcentral gyrus, and bilateral cerebellum posterior lobe depending on PE direction. We observed higher ALFF values in the right precentral and postcentral gyri and lower ALFF values in the bilateral cerebellum posterior lobe in males than in females regardless of PE direction as indicated by the main effect of gender. The precentral and postcentral gyri are associated with sensorimotor integration, and gender difference in sensorimotor characteristics also has been revealed (Crozier et al., 2019). However, when we examined gender differences in these brain areas, we only found these differences in the PA direction. In addition, gender differences in ReHo differed remarkably in the right lingual gyrus depending on PE direction. The lingual gyrus is a brain region involved in higher-order visual processing and mental imagery (Landry et al., 2017). Previous studies showed that females are better at inspecting mental images than males (Isaac and Marks, 1994; Palermo et al., 2016). In our study, females showed significantly higher ReHo in lingual gyrus than males for AP imaging, but no gender difference was found for PA imaging. Additionally, we also found remarkable interaction effect between gender and PE direction (LR and RL) in several brain regions for all the indices except the rsFC of PCC by applying the HCP data, which further verified that PE direction in fMRI can affect the results of gender differences in estimation of spontaneous brain activity in rsfMRI. Although the brain regions of the interaction effects found in these two datasets were not completely consistent, the results mutually indicated that the choice of PE direction will affect the results of group analysis. Therefore, the conclusions drawn from the group analyses for the data acquired from the same participants but with different PE directions are not in agreement. Based on the above analyses, we can see that the choice of PE direction in fMRI can influence the conclusions on group differences. Future studies of these areas might benefit from the careful choice of PE direction because of the increased sensitivity to group differences.

There are several limitations to this study. First, we did not balance the collection order of rsfMRI imaging for different PE directions. The two sets of fMRI scans were acquired in the same order: PA first and then AP in our dataset. In the HCP dataset, the collection order of rsfMRI imaging for different PE directions (LR and RL) was counterbalanced across participants and we still found remarkable interaction effect between gender and PE direction in several brain regions. Therefore, the potential order effect may have little impact on the current findings. Second, females usually have smaller brain size than males (Ruigrok et al., 2014); thus, the susceptibility gradient-induced signal loss and geometrical distortion may be different between males and females. This may confound the present results on gender differences. Third, we only selected several commonly used functional indices, including ALFF, fALFF, ReHo, DC, and seed-based rsFC, and did not conduct other rsfMRI analysis, such as ICA, which can estimate the connectivity of large-scale networks. Future studies that use different methods would be informative. Finally, we did the susceptibility artifact correction for the functional images by using diffusion MRI images, which may not be a perfect approach. It is recommended to conduct susceptibility artifact corrections by collecting spin-echo EPI images for fMRI data (Smith et al., 2013). Conventional approach that estimates the displacement field based on reversed PE image pairs is time-consuming in data collection (Hutton et al., 2002; Holland et al., 2010). Recently, Togo et al. (2017) reported that distortion correction with field map improves the accuracy of registration and further improves the quality of both task and resting-state fMRI. Duong et al. (2020b) proposed an unsupervised deep learning technique for susceptibility artifact correction (S-Net), which accelerates the medical image processing pipelines and makes the real-time correction for MRI scanners feasible (Duong et al., 2020b). However, this process may be difficult to perform because of the scanning time limit and technical difficulties, especially in a clinical context. Under these constraints, researchers need to interpret the findings carefully and pay close attention to the brain regions that might be affected by PE direction. We encourage researchers to choose the appropriate PE direction based on the brain areas they focus on if the conditions are limited. Considering that the scans of the two PE directions had signal loss in different brain regions (Figure 1), PA direction would be recommended if researchers are more concerned about the orbital gyrus, middle TG, and anterior cingulate gyrus and AP direction would be recommended if researchers are more concerned about the gyrus rectus, inferior TG, caudate, and cerebellum than other brain regions.

## Conclusion

Choosing a different PE direction was confirmed to partially affect gender differences in estimation of spontaneous brain activity in rsfMRI. These apparent findings deepen our understanding of how PE directions in fMRI can affect the findings of group studies. Researchers need to be more cautious when reporting and interpreting the findings if the neuroimaging data is collected from a single PE direction. Future rsfMRI studies should specify the adopted PE direction, and appropriate selection of PE direction will be important in future rsfMRI studies.

## Data Availability Statement

The raw data supporting the conclusions of this article will be made available by the authors, without undue reservation.

## Ethics Statement

The studies involving human participants were reviewed and approved by the Ethics Committee of Beijing Anding Hospital of Capital Medical University. The patients/participants provided their written informed consent to participate in this study.

## Author Contributions

YZ and GW: conception or design of the work. YW, XC, RL, ZZ, JZ, YF, and CJ: data collection and data curation. YW and YZ: data analysis and interpretation. YW: drafting the article. YZ, GW, and X-NZ: critical revision of the article. All authors read and approved the final manuscript.

## Conflict of Interest

The authors declare that the research was conducted in the absence of any commercial or financial relationships that could be construed as a potential conflict of interest.

## Publisher’s Note

All claims expressed in this article are solely those of the authors and do not necessarily represent those of their affiliated organizations, or those of the publisher, the editors and the reviewers. Any product that may be evaluated in this article, or claim that may be made by its manufacturer, is not guaranteed or endorsed by the publisher.

## Footnotes

1
http://www.restfmri.net

2
http://www.fil.ion.ucl.ac.uk/spm
