# Supplementary material for: Effect of Phase-Encoding Direction on Gender Differences: A Resting-State Functional Magnetic Resonance Imaging Study
Source: Front Neurosci. 2022 Jan 25;15:748080. doi: 10.3389/fnins.2021.748080 (PMC8824585; doi:10.3389/fnins.2021.748080)
Supplement: Supplementary file 1 [file Data_Sheet_1.PDF]

Supplementary Materials

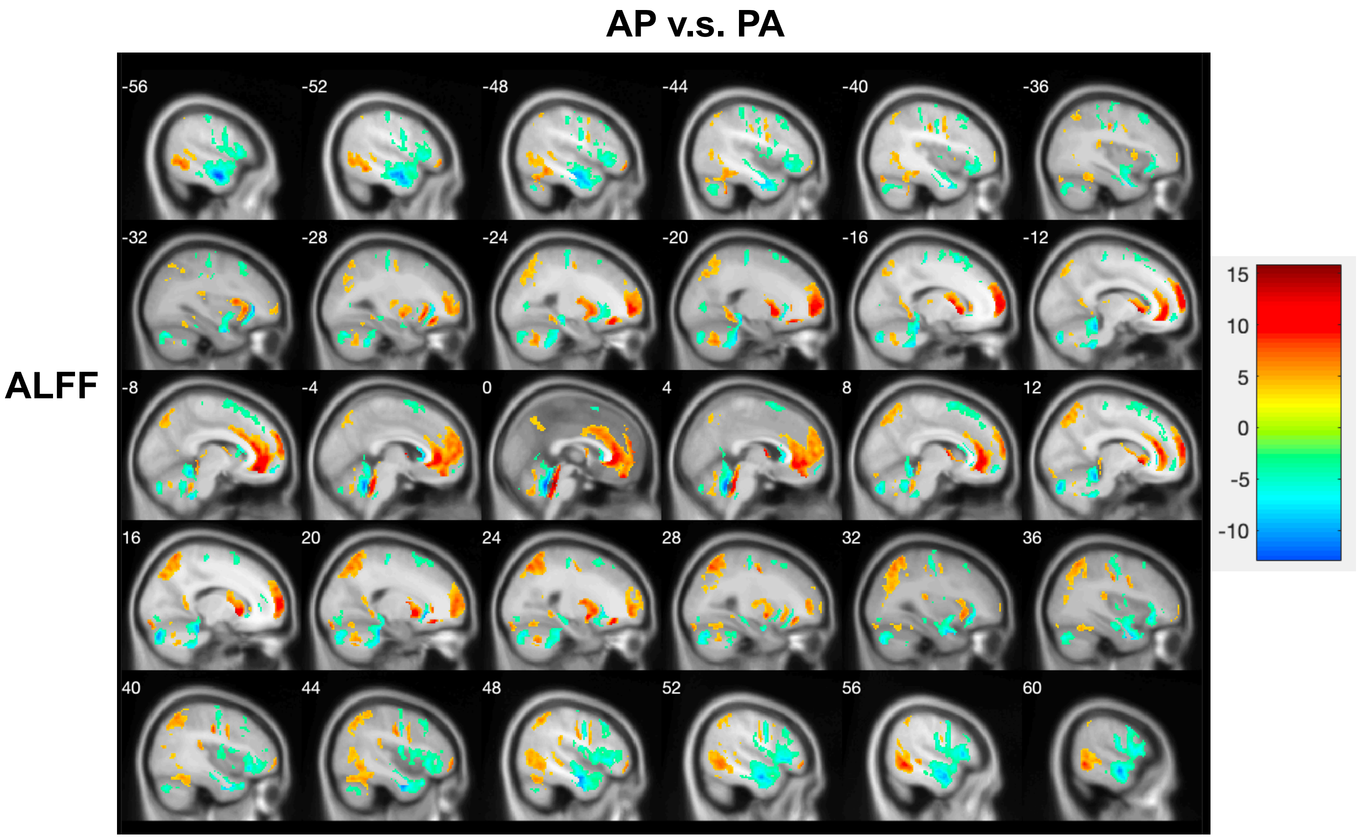

Figure S1. Regions that showed different ALFF values depending on phase-encoding direction. Red indicates regions where the AP direction estimated significantly greater brain activities than the PA direction. Blue indicates the reverse.

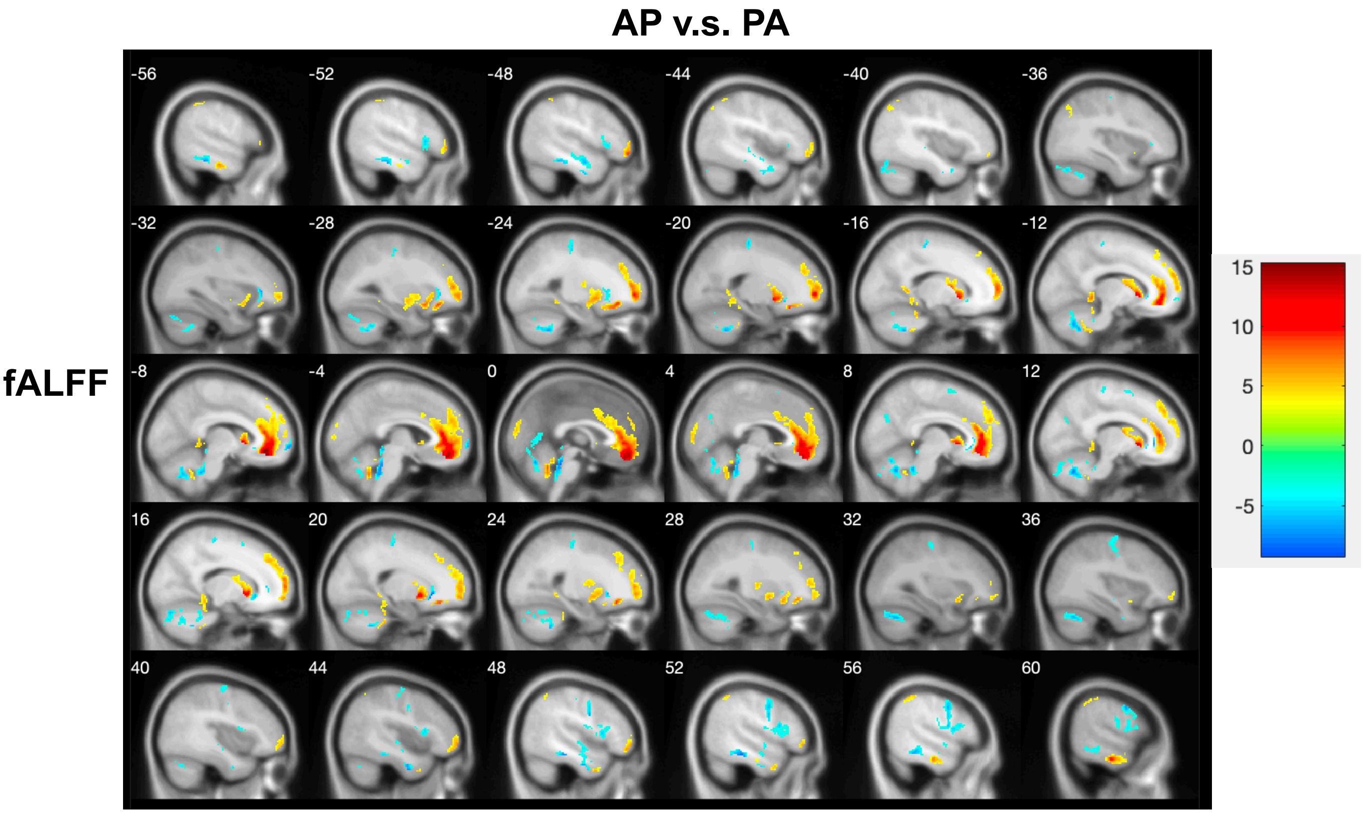

Figure S2. Regions that showed different fALFF values depending on phase-encoding direction. Red indicates regions where the AP direction estimated significantly greater brain activities than the PA direction. Blue indicates the reverse.

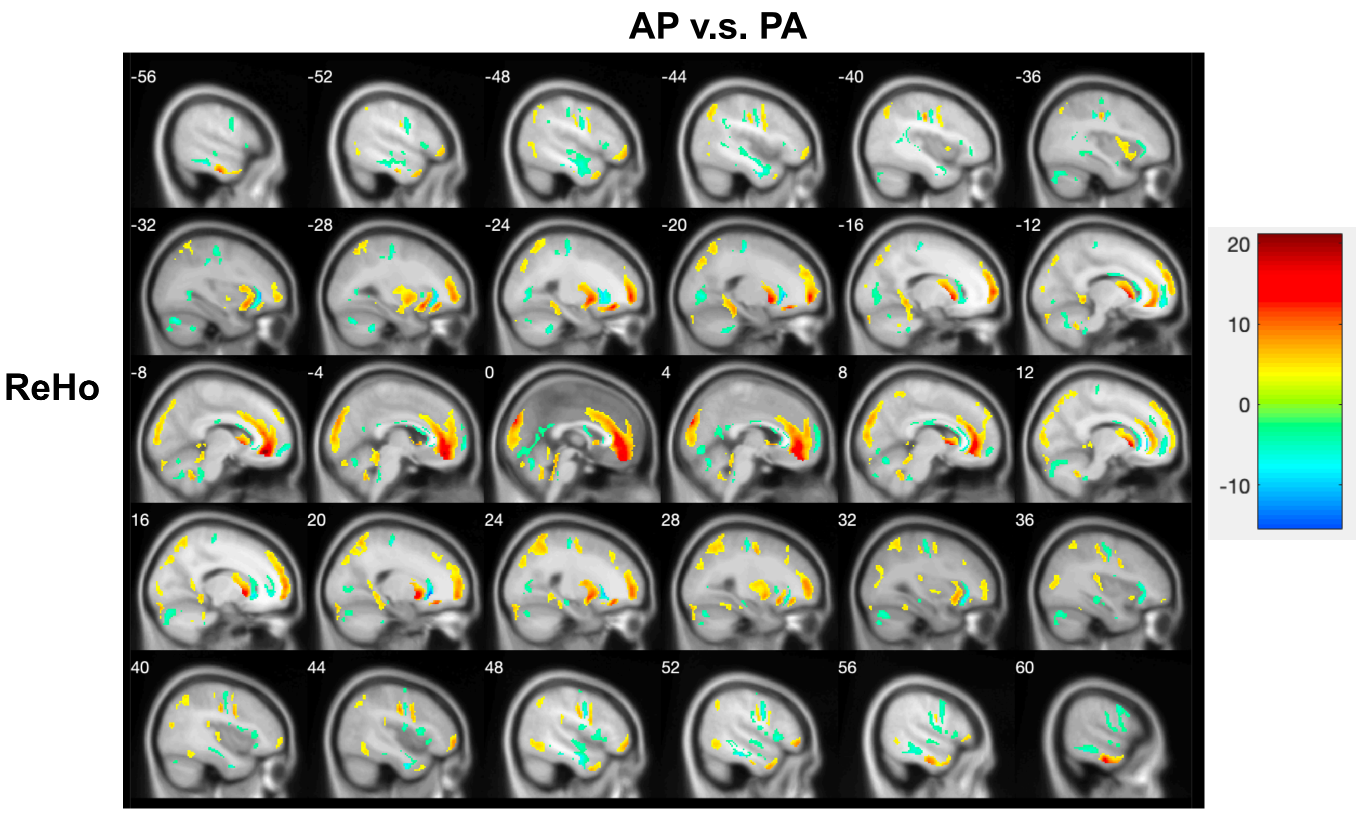

Figure S3. Regions that showed different ReHo values depending on phase-encoding direction. Red indicates regions where the AP direction estimated significantly greater brain activities than the PA direction. Blue indicates the reverse.

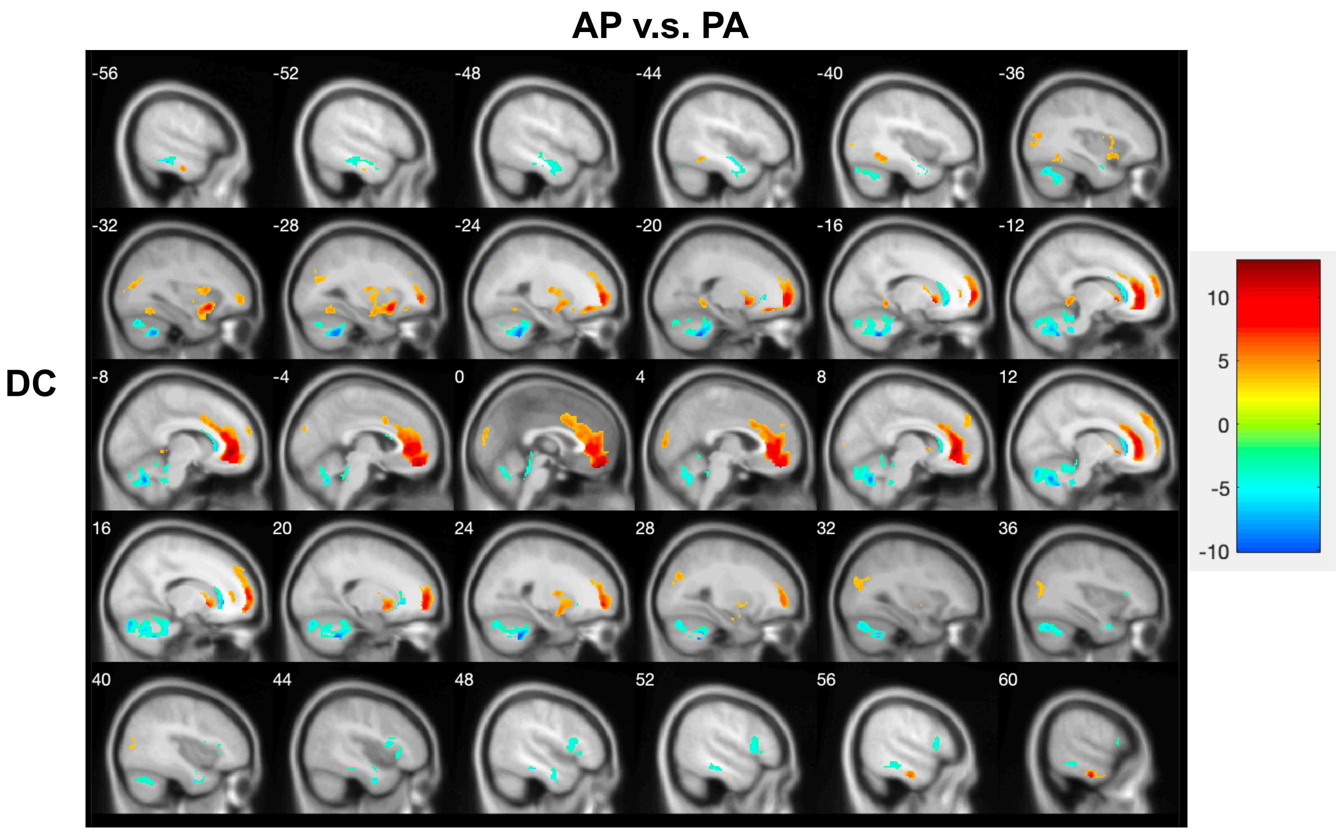

Figure S4. Regions that showed different DC values depending on phase-encoding direction. Red indicates regions where the AP direction estimated significantly greater brain activities than the PA direction. Blue indicates the reverse.

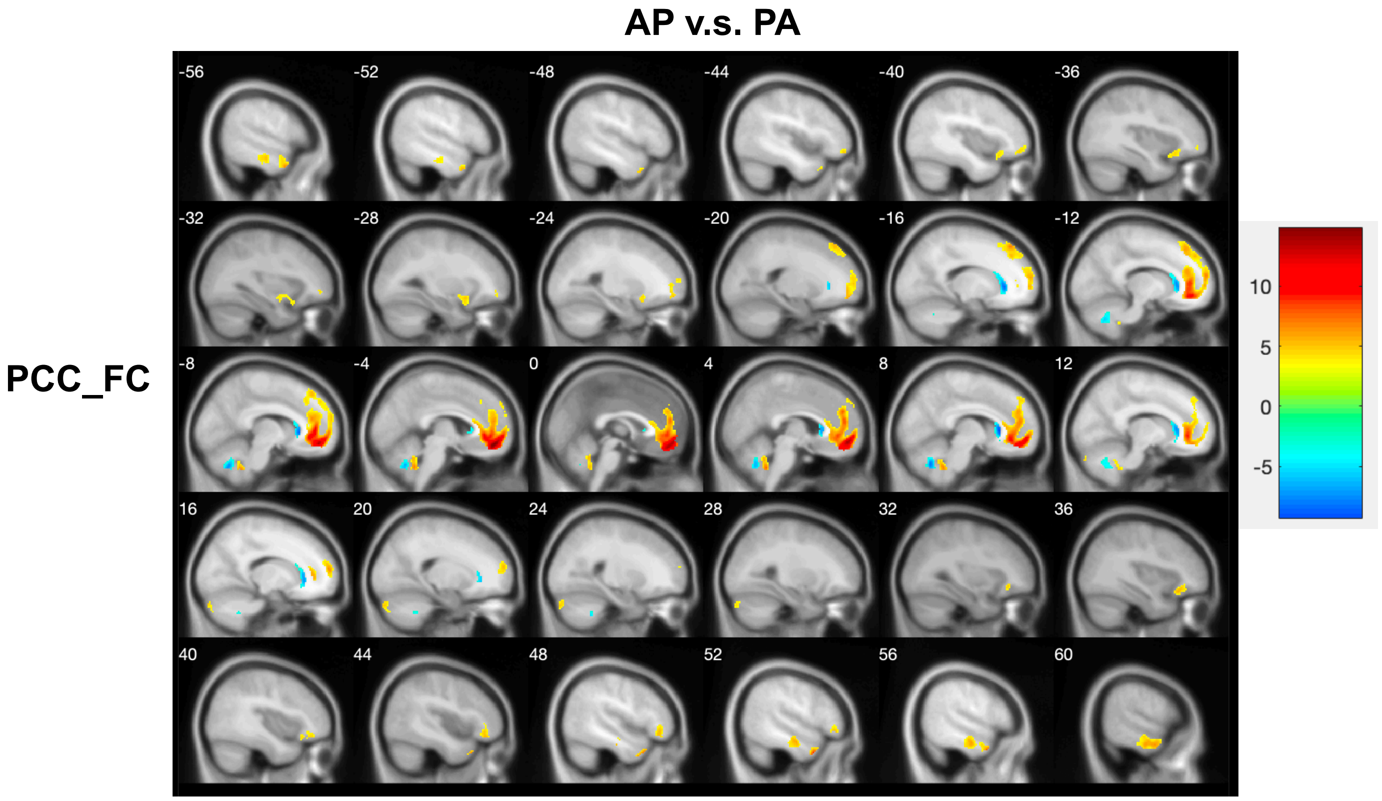

Figure S5. Regions that showed different FCs with the PCC depending on phase-encoding direction. Red indicates regions where the AP direction estimated significantly greater functional connections with the PCC than the PA direction. Blue indicates the reverse.

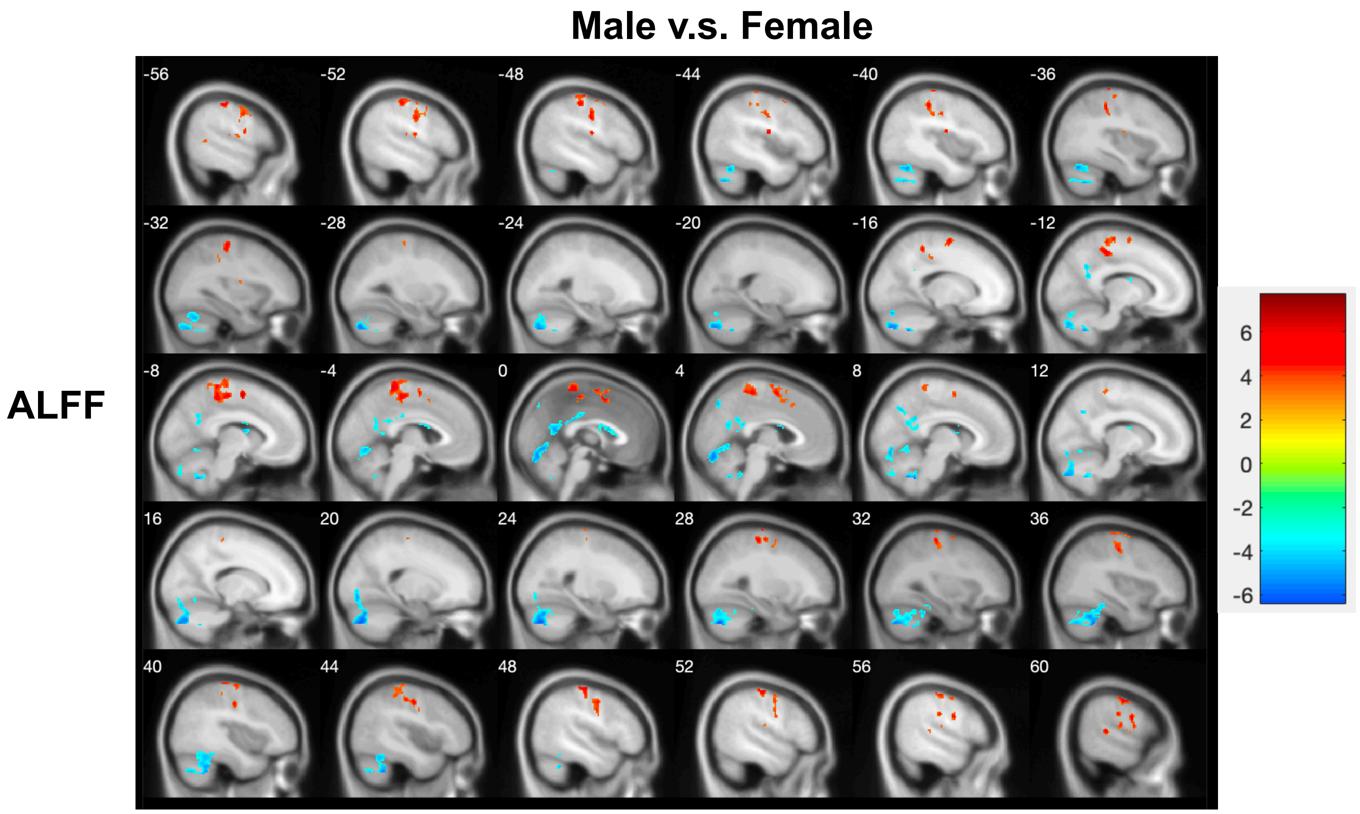

Figure S6. Regions that showed different ALFF values between males and females. Red indicates regions where the males showed significantly greater brain activities than the females. Blue indicates the reverse.

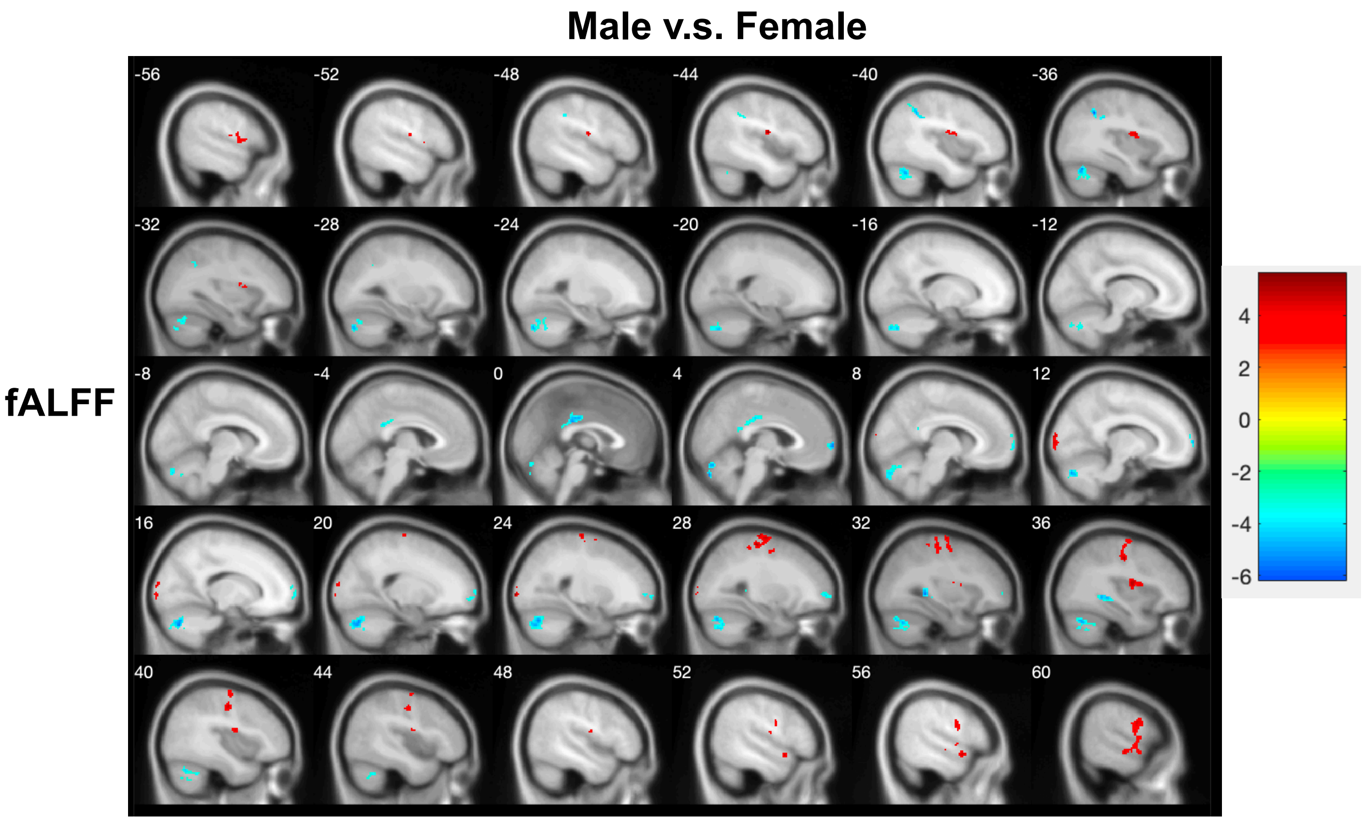

Figure S7. Regions that showed different fALFF values between males and females. Red indicates regions where the males showed significantly greater brain activities than the females. Blue indicates the reverse.

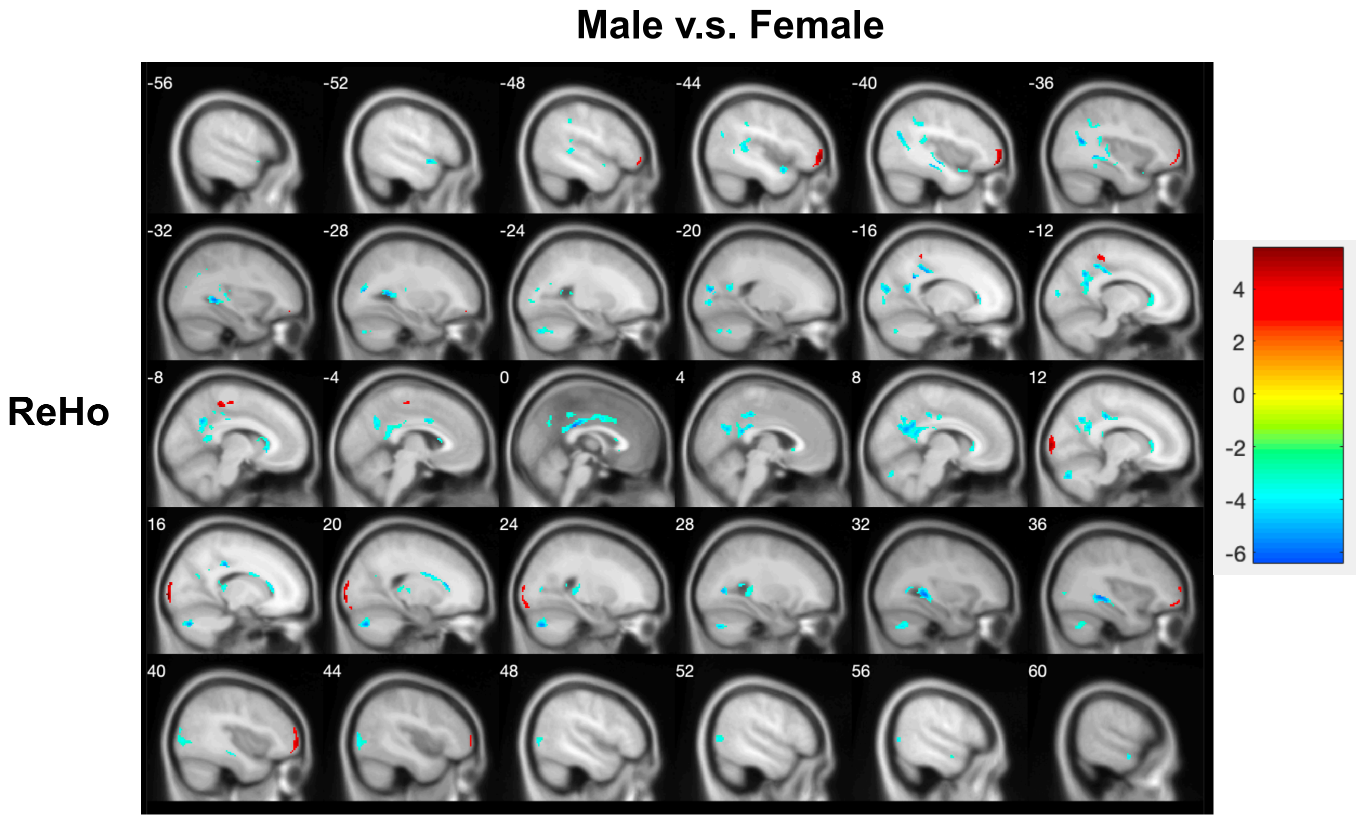

Figure S8. Regions that showed different ReHo values between males and females. Red indicates regions where the males showed significantly greater brain activities than the females. Blue indicates the reverse.

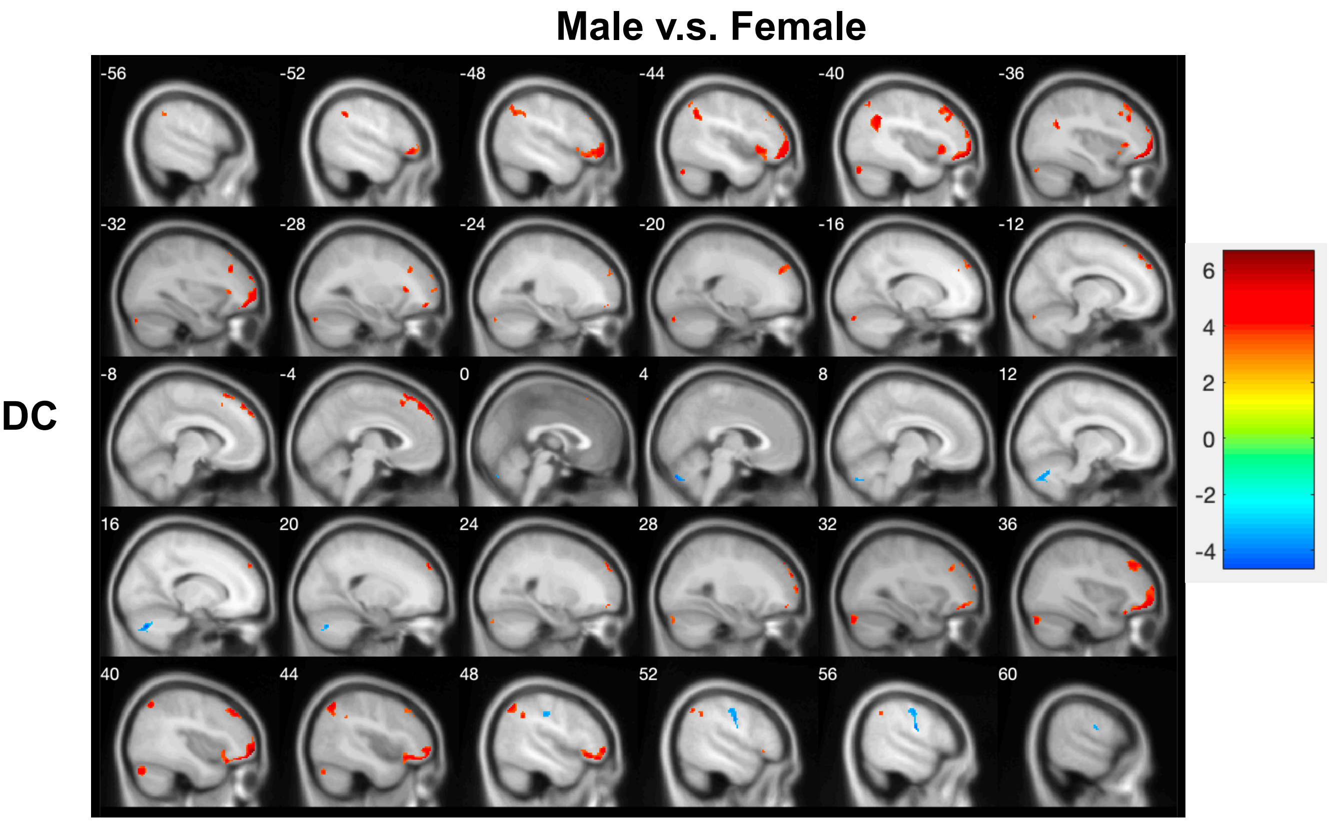

Figure S9. Regions that showed different DC values between males and females. Red indicates regions where the males showed significantly greater brain activities than the females. Blue indicates the reverse.

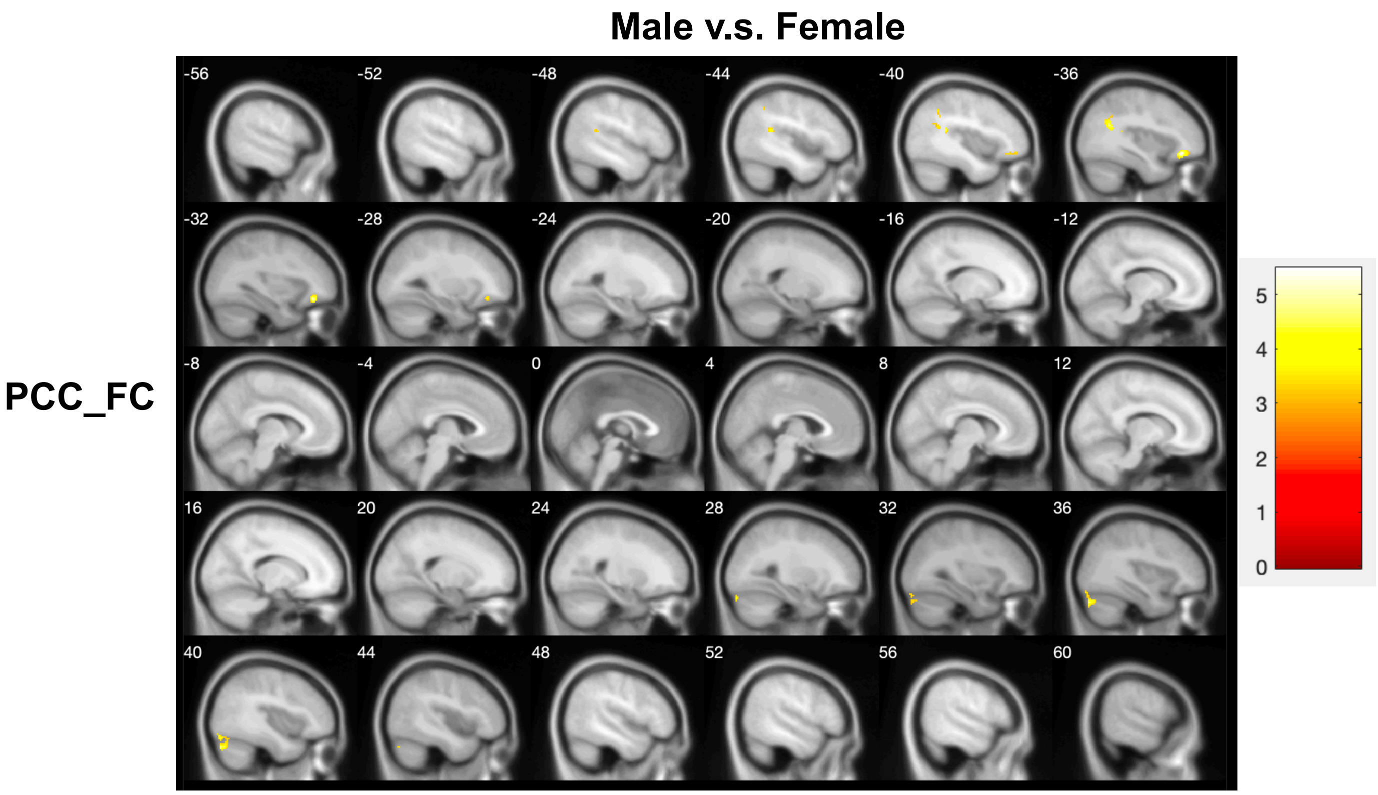

Figure S10. Regions that showed different FCs with the PCC between males and females. Red indicates regions where the males showed significantly greater functional connections with the PCC than the females.

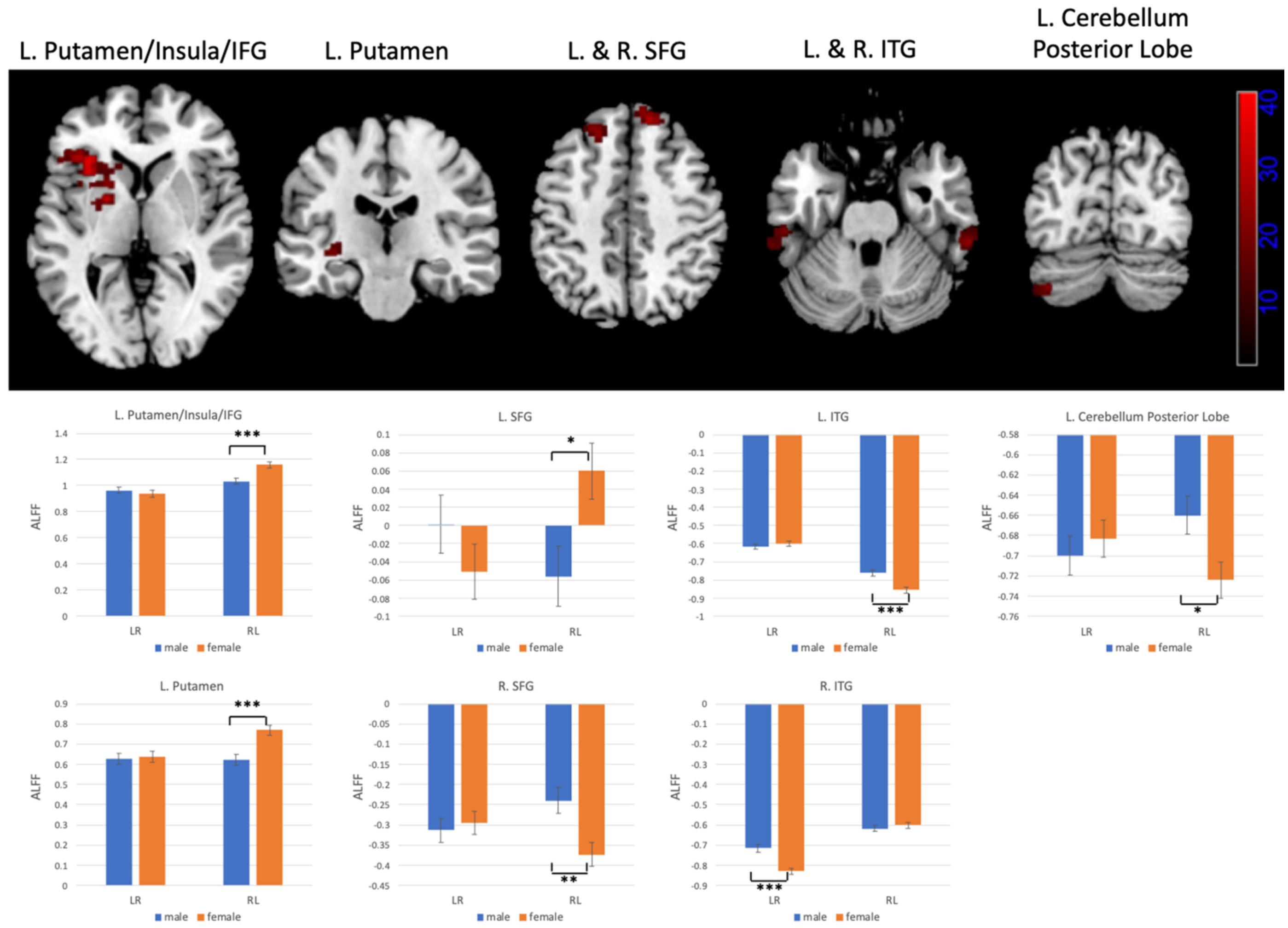

Figure S11. Regions influenced by the interaction effect between gender and PE direction (LR v.s. RL) for ALFF in the HCP data and the results of post hoc analysis. Abbreviation: IFG, inferior frontal gyrus; SFG, superior frontal gyrus; ITG, inferior temporal gyrus. *$p < 0.05$, **$p < 0.01$, ***$p < 0.001$.

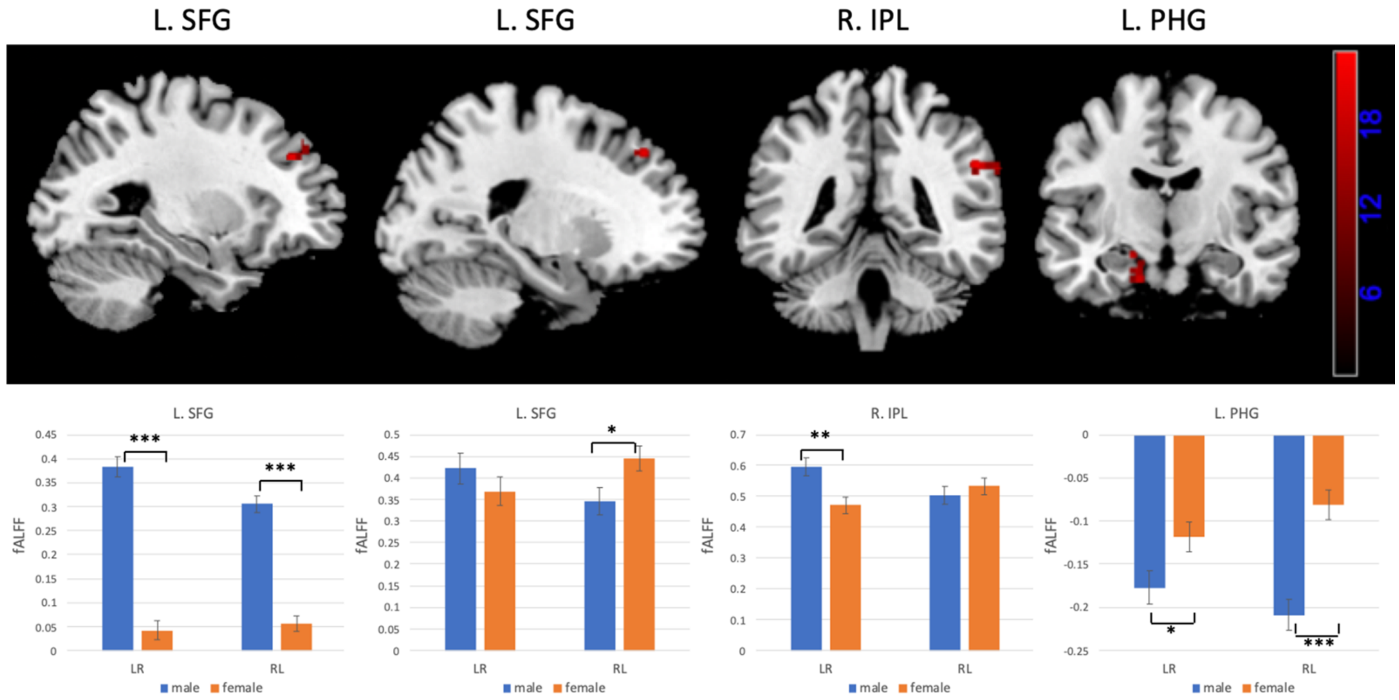

Figure S12. Regions influenced by the interaction effect between gender and PE direction (LR v.s. RL) for fALFF in the HCP data and the results of post hoc analysis. Abbreviation: SFG, superior frontal gyrus; IPL, inferior parietal lobule; PHG, parahippocampa gyrus. *$p < 0.05$, **$p < 0.01$, ***$p < 0.001$.

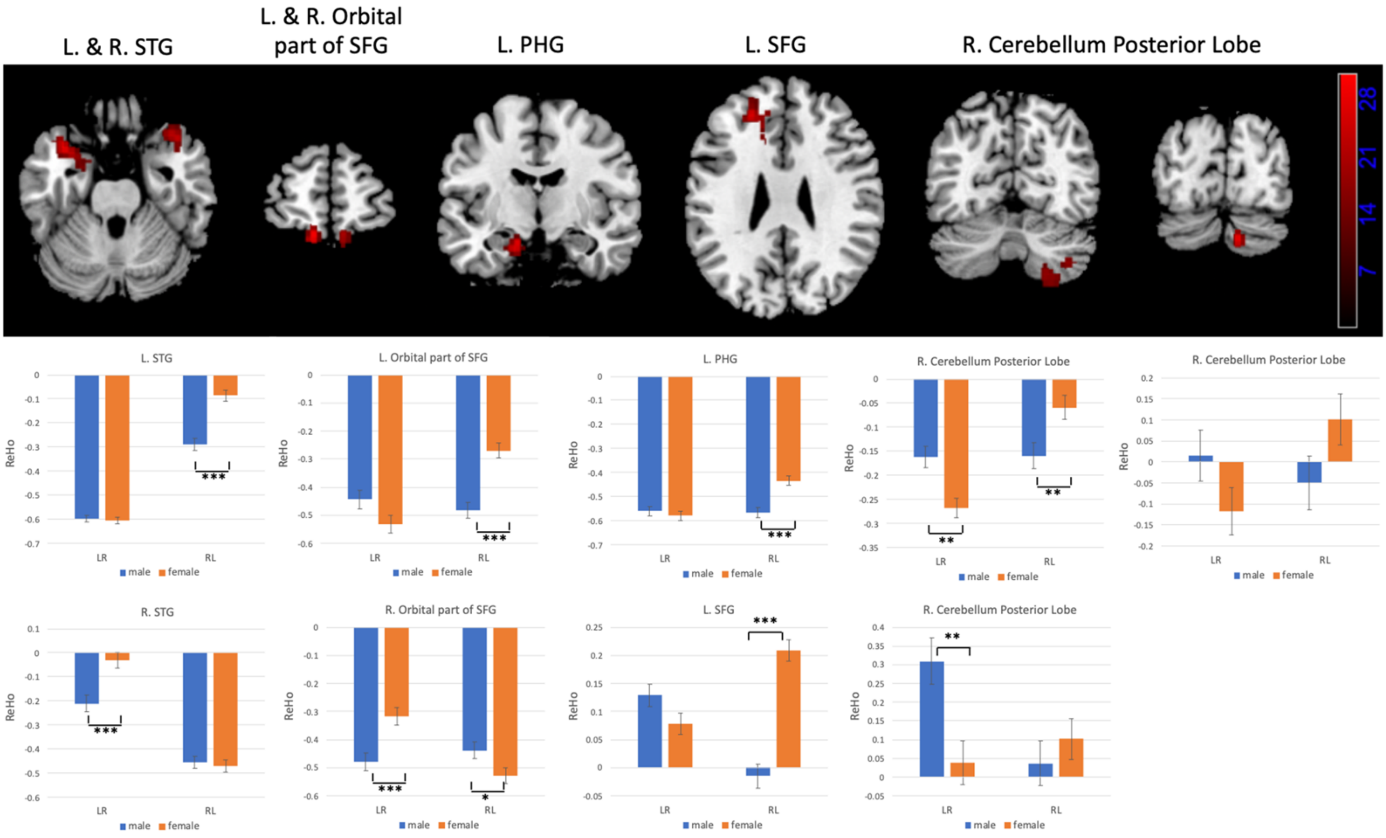

Figure S13. Regions influenced by the interaction effect between gender and PE direction (LR v.s. RL) for ReHo in the HCP data and the results of post hoc analysis. Abbreviation: STG, superior temporal gyrus; SFG, superior frontal gyrus; PHG, parahippocampa gyrus. *$p < 0.05$, **$p < 0.01$, ***$p < 0.001$.

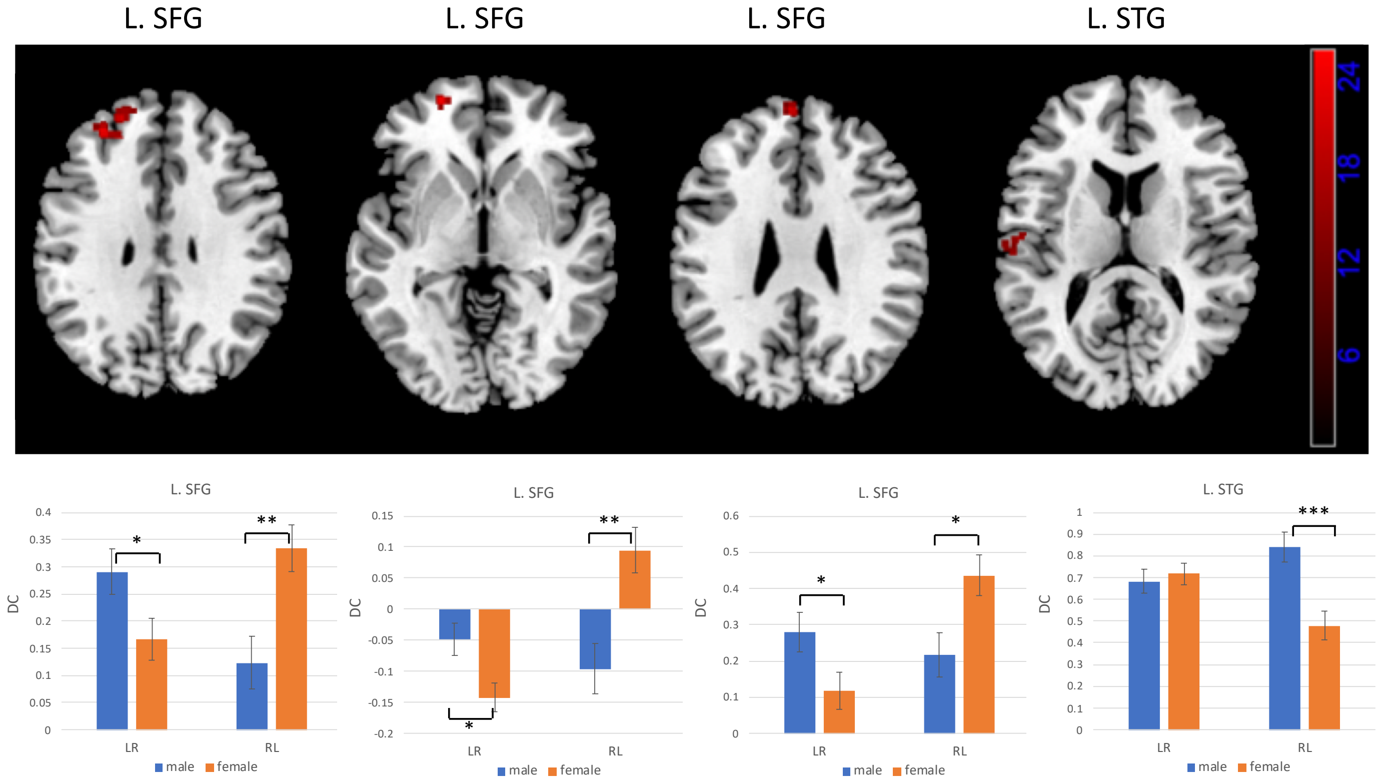

Figure S14. Regions influenced by the interaction effect between gender and PE direction (LR v.s. RL) for DC in the HCP data and the results of post hoc analysis. Abbreviation: SFG, superior frontal gyrus; STG, superior temporal gyrus. *$p < 0.05$, **$p < 0.01$, ***$p < 0.001$.
